# The effectiveness of dexmedetomidine for preventing acute kidney injury after surgery: a systematic review and meta-analysis

**DOI:** 10.3389/fmed.2024.1414794

**Published:** 2024-05-24

**Authors:** Jing Zhao, Ming-hao Tang, Qi-hong Shen, Ding-chao Xu

**Affiliations:** ^1^Department of Anesthesiology, Jiashan First People’s Hospital, Jiaxing, China; ^2^Department of Anesthesiology, First Hospital of Jiaxing, Jiaxing, China

**Keywords:** acute kidney injury, dexmedetomidine, meta-analysis, postoperative delirium, bradycardia

## Abstract

**Background:**

Postoperative acute kidney injury (AKI) is a serious and distressing complication connected to various adverse outcomes following the surgical operation. Controversy remains regarding the dexmedetomidine’s preventive impact on postoperative AKI. Therefore, this investigation aims to explore the efficiency and safety of dexmedetomidine in preventing AKI after surgical operation.

**Methods:**

We systematically searched electronic databases such as PubMed, Embase, Web of Science, and the Cochrane Library to detect eligible randomized controlled studies that used dexmedetomidine for the prevention of AKI following operation up to April 30, 2023. The main outcome evaluated was AKI incidence. The evidence quality was assessed employing the Grading of Recommendations Assessment, Development, and Evaluation.

**Results:**

The meta-analysis included 25 trials, including 3,997 individuals. Of these, 2,028 were in the dexmedetomidine group, and 1,969 were in the control group. The result showed that patients administered dexmedetomidine significantly decreased the AKI incidence following surgical operation in contrast to the control group (risk ratio, 0.60; 95% confidence intervals, 0.45–0.78; *p* < 0.05; *I*^2^ = 46%). In addition, dexmedetomidine decreased the period of hospitalization in both the intensive care unit (ICU) and the hospital while also reducing postoperative delirium (POD) occurrence. However, dexmedetomidine elevated the incidence of bradycardia but did not have a significant impact on other indicators.

**Conclusion:**

Our meta-analysis indicates that the dexmedetomidine treatment reduces the postoperative AKI and POD risk while also shortening the time of hospitalization in the ICU and hospital. However, it is connected to an increased bradycardia risk.

## Introduction

Acute kidney injury (AKI) is a significant postoperative complication, resulting in a prolonged stay in the hospital, increased hospitalization costs, and death. The AKI occurrence varies according to the specific surgical procedure. Studies have indicated that AKI incidence following a cardiac operation ranged from 20 to 70% (1, 2), while it ranged from 6.1 to 22.4% after major non-cardiac surgeries depending on the type of surgery (3–7). The development of postoperative AKI is influenced by multiple factors, with research suggesting a close association with oxidative stress, inflammatory response, sympathetic nervous system activation, and injury with ischemic reperfusion (8). Consequently, identifying effective intervention measures and optimizing perioperative medication is crucial.

Dexmedetomidine, a highly specific α2-adrenergic agonist, is often employed to induce sedation and relieve pain during the anesthetic process. Studies have demonstrated that dexmedetomidine can mitigate ischemia–reperfusion injury by reducing oxidative stress (9). Recent clinical investigations have also suggested dexmedetomidine’s potential protective role in reducing AKI incidence (10, 11). However, there is still controversy over the protective effect of dexmedetomidine on renal function. Hence, we performed this meta-analysis to explore dexmedetomidine efficiency and safety in preventing postoperative AKI.

## Methods

The Preferred Reporting Items for Systematic Reviews and Meta-Analysis standards were employed to perform and document this systematic review and meta-analysis (12). The investigation was recorded under the number registration CRD42023397747 with the International Prospective Register of Systematic Reviews.

### Systematic literature search

“PubMed,” EMBASE,” “Cochrane Library,” and “Web of Science,” electronic databases, were employed to perform a systematic search. The literature screening method included investigations from the beginning of these databases until April 30, 2023, without any limitations on language. The search strategies employed for each electronic database are provided in the Supplementary materials. Furthermore, the reference lists of the determined papers were systematically reviewed and analyzed.

### Inclusion and exclusion criteria

The investigation’s inclusion criteria were: (1) Participants (P): subjects who have had surgical procedures; (2) Intervention (I): dexmedetomidine administration; (3) Comparison (C): placebo or other pharmacological therapies; (4) The outcomes (O): studies that record the occurrence of AKI; and (5) design of the study (S): RCTs. The exclusion criteria included (1) individuals who did not get surgical treatment, (2) patients who were not receiving operations, (3) current investigations, and (4) conference abstracts.

### Extraction of data and outcomes

First, two authors independently employed EndNote to identify and eliminate duplicate investigations. Subsequently, the title and abstract were assessed to detect if they met the inclusion criteria. Finally, a comprehensive examination of the full text of these articles was performed to assess their eligibility for inclusion in the study. Two authors independently obtained and verified additional data from the chosen papers: the authors’ names, publication year, kind of surgical procedure conducted, sample size, anesthetic specifics, dexmedetomidine dosages, and evaluation techniques for AKI. The primary finding measure of the investigation was to determine the occurrence of AKI. The definition of AKI was based on the authors of each included trial. Other factors that were also examined as secondary results were the time length of connecting to a mechanical ventilator, the period of their presence in the intensive care unit (ICU) and hospital, in addition to the occurrence of postoperative delirium (POD), death, bradycardia, stroke, and hypotension.

### Evaluation of the quality and risk

To evaluate the potential for bias in the investigations that were comprised, the Cochrane Collaboration method was utilized. The evaluation of bias covered the following areas: biased selection (random sequence generation and distribution concealment), detection bias (blinding of results assessors), performance bias (blinding of the subjects and personnel), attrition bias (incomplete results reporting), reporting bias (selective outcome reporting), and other biases. Every study was classified as either having an elevated bias risk, moderate concerns, or a low risk of bias. Additionally, the confidence level in the evidence was assessed by employing the Grading of Recommendations Assessment, Development, and Evaluation (GRADE) approach. The certainty of the included studies was classified as high, moderate, low, or very low.

### Statistical analysis

Meta-analysis was conducted utilizing two statistical programs: “Review Manager 5.3” (version 5.3, Copenhagen) and “Stata version 12.0” (Stata Corp. LP, United States). The incidence of POD was assessed by calculating the combined risk ratio (RR) and related 95% confidence intervals (CIs). A *p*-value of <0.05 was considered statistically significant. The heterogeneity across the studies comprised in the investigation was evaluated employing the *I*^2^ statistic, where values over 50% indicate a high level of heterogeneity. Clinical and methodological factors were identified as potential sources of heterogeneity. Therefore, the random-effects model was employed for investigations with low *I*^2^ values.

Subgroup analyses were conducted based on surgery types (cardiac vs. non-cardiac surgery) and different age groups (children vs. adults). We used TSA program (0.9.5.10 beta version) to reduce the risk of Type I errors resulting from repeated testing. TSA provides boundaries and required information size lines. If the cumulative *z*-curve fails to cross the border of monitoring or reach the appropriate information size line, more investigation is deemed essential.

## Results

### Search results

**Figure 1:**
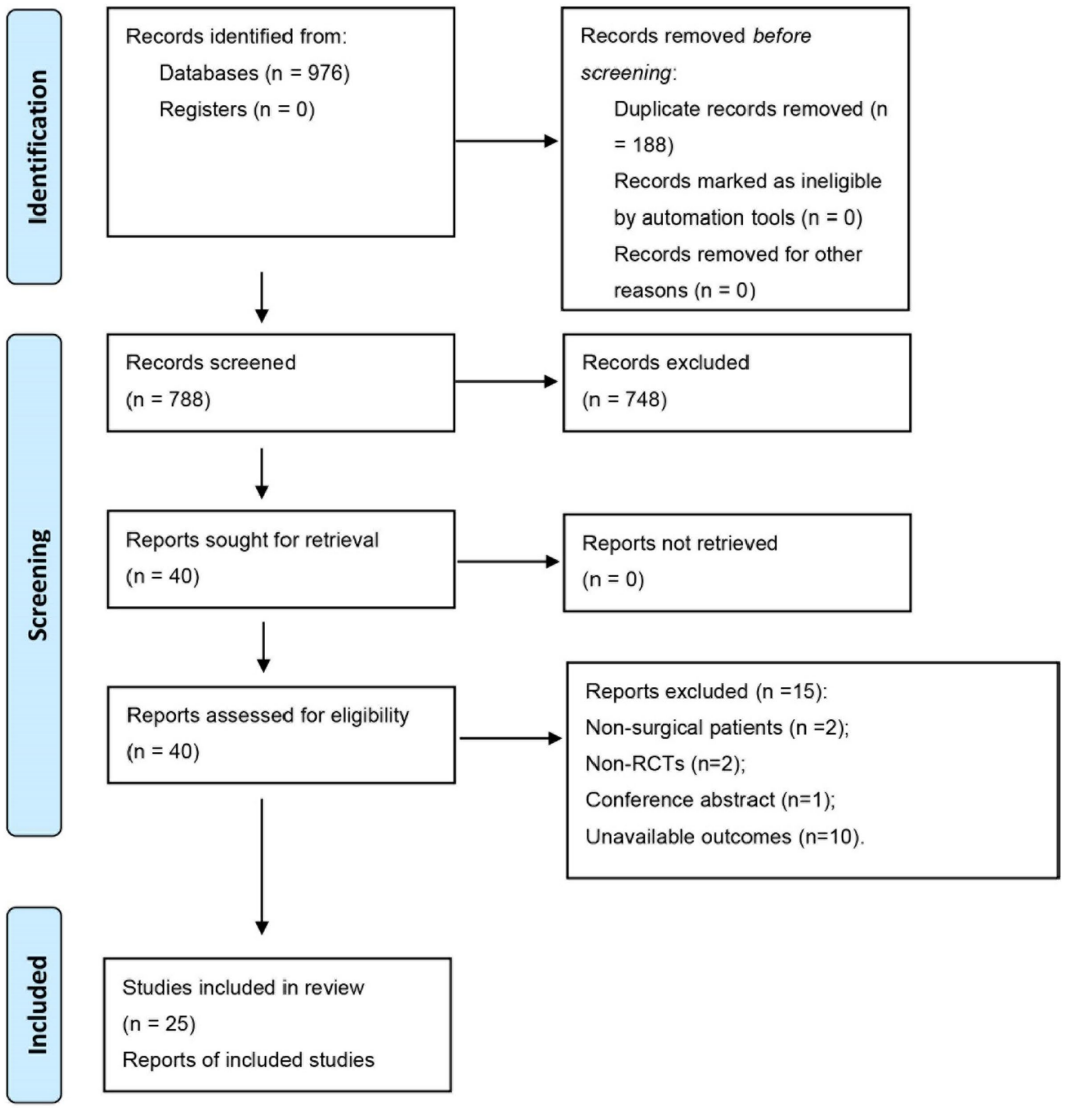
A flow chart for literature inclusion.

Initially, 976 investigations were examined from electronic databases through systematic searches. Subsequently, 188 duplicate studies were deleted. Subsequently, 619 studies were further excluded through the title and abstract. Subsequently, a thorough reading and analysis of the remaining 40 papers were conducted to detect whether they were ultimately comprised. Among these, 15 articles were excluded for various reasons, including non-surgical patients (*n* = 2), non-RCTs (*n* = 2), conference abstracts (*n* = 1), and unavailable outcomes (*n* = 10). Ultimately, 25 manuscripts met the inclusion criteria (10, 11, 13–35). Figure 1 illustrates the process of literature screening.

### Study characteristics

The comprised articles ranged in publication year from 2009 to 2023, with sample sizes varying from 29 to 619 patients. Three studies specifically focused on pediatric patients (23, 30, 32), while the remaining studies involved adult patients. All studies did not include patients diagnosed with chronic kidney disease. Table 1 illustrates more detailed data on these investigations.

**Table 1:** The details of included studies.

Study	Sample size	Age	Type of surgery	Anesthesia induction	Anesthesia maintenance	Dex group	Control group	Definition of AKI
Ammar et al. (18)	Dex: 25 Control: 25	Adult	Cardiac surgery	Propofol 1.5–2 mg/kg, fentanyl 3–5 μg/kg, cis-atracurium 0.1 mg/kg	Isoflurane at 1.0 MAC, fentanyl 3–5 μg/kg/h, and cis-atracurium 2 μg/kg/min	1 μg/kg over 15 min, followed by 0.5 μg/kg/h	Saline	NR
Balkanay et al. (17)5	Dex: 31 + 29 Control: 28	Adult	Cardiac surgery	NR	NR	0.04–0.5 μg/kg/h	Saline	RIFLE
Cho et al. (19)	Dex: 100 Control: 100	Adult	Cardiac surgery	NR	NR	0.4 μg/kg/h	Saline	AKIN
Deng et al. (27)	Dex: 95 Control: 95	20–75	Postpercutaneous nephrolithotomy lithotripsy	Midazolam 0.035 mg/kg, fentanyl 3 μg/kg, propofol 1.5–2 mg/kg, cis-atracurium 0.2 mg/kg	Sevoflurane 2–2.5%	1 μg/kg for 15 min, followed by 0.5 μg/kg/h	Saline	NR
Djaiani et al. (20)	Dex: 91 Control: 92	> 70	Cardiac surgery	Fentanyl 10–12 μg/kg, propofol 0.5–2 mg/kg, pancuronium 0.15 mg/kg	Isoflurane 0.5–2.0%	0.4 μg/kg over 10–20 min, followed by 0.2–0.7 μg/kg/h	Propofol 2–5 μg/kg/min until extubation	NR
Jo et al. (23)	Dex: 15 Control: 14	1–6	Cardiac surgery	Ketamine 1–2 mg/kg, sufentanil 1–2 mg/kg, rocuronium of 0.8 mg /kg	Sevoflurane 2–3%	0.5 μg/kg for 10 min, followed by 0.5 μg/kg/h	Saline	AKIN
Kim et al. (30)	Dex: 71 Control: 68	<7	Cardiac surgery	Thiopental sodium 5 mg/kg, fentanyl 2–3 μg/kg	Sevoflurane 2–3%, remifentanil 0.2 μg/ kg/min	1 μg/kg over 10 min, followed by 0.5 μg/kg/h	Saline	KDIGO
Leino et al. (14)	Dex: 35 Control: 31	>21	Cardiac surgery	Fentanyl 30 μg/kg, lorazepam 2 mg, pancuronium 0.1 mg/kg	Fentanyl 0.1 μg/kg/min, isoflurane	Steady-state plasma concentration of 0.60 ng/mL	Saline	RIFLE
Li et al. (24)	Dex: 142 Control: 143	≥ 60	Cardiac surgery	Midazolam, etomidate, sufentanil, propofol	Sufentanil, propofol and/or sevoflurane	0.6 μg/kg for 10 min, followed by 0.4 μg/kg/h	Saline	KDIGO
Liu et al. (21)	Dex: 44 Control: 44	≥18	Cardiac surgery	Midazolam, sufentanil, cis-atracurium	Sevoflurane, propofol, sufentanil	≤ 1.5 μg/kg/h	propofol ≤3 mg/kg/h	AKIN
Ming et al. (32)	Dex: 30 + 30 Control: 30	1–6	Cardiac surgery	Vecuronium bromide 0.1 mg/kg, fentanyl 3–8 g/kg, propofol 1–3 mg/kg	NR	0.2 μg/kg/h or 0.4 μg/kg/h	Saline	Kidney Disease
Park et al. (15)	Dex: 67 Control: 75	18–89	Cardiac surgery	Propofol, fentanyl, rocuronium	Fentanyl, inhalation agents	0.5 μg/kg for loading does, 0.2–0.8 μg/kg/h	Remifentail 1,000–2,500 μg/h	NR
Shan et al. (10)	Dex: 48 Control: 50	≥ 18	Cardiac surgery	Propofol 1.5 mg/kg, sufentanil 0.4 μg/kg, cis-atracurium 0.2 mg/kg	Sevoflurane 1–3%	0.4 μg/kg/h during surgery, 0.1 μg/kg/h in the ICU	Saline	AKIN
Shehabi et al. (13)	Dex: 152 Control: 147	≥ 60	Cardiac surgery	Midazolam 0.1–0.15 mg/kg, fentanyl 15–25 μg/kg, pancuronium 0.2 mg/kg	Sevoflurane 2–3%	0.1–0.7 μg/kg/ml	Morphine 10–70 μg/kg/ml	NR
Soh et al. (31)	Dex: 54 Control: 54	≥ 20	Cardiac surgery	Midazolam 0.05 mg/kg, sufentanil 1.5 μg/kg	Sevoflurane, sufentanil	0.4 μg/kg/h	Saline	KDIGO
Soliman and Hussien(25)	Dex: 75 Control: 75	Adult	Cardiac surgery	Midazolam 0.03–0.1 mg/kg, fentanyl 3–5 μg/kg, etomidate 0.3 mg/kg, rocuronium 0.6 mg/kg	Sevoflurane 1–3%, fentanyl 1–3 μg/kg/h, cis-atracurium 1–2 μg/kg/min	0.4 μg/kg/h	Saline	NR
Soliman and Zohry (22)	Dex: 75 Control: 75	Adult	Cardiac surgery	Fentanyl 1–2 μg/kg, thiopental 3–5 mg/kg, atracurium 0.5 mg/kg	Sevoflurane 1–3%, fentanyl 1–3 μg/kg/h, atracurium (0.5 mg/kg/h	1 μg/kg over 15 min, followed by 0.3 μg/kg/h	Saline	Creatinine >115 μmol/L
Song et al. (28)	Dex: 19 Control: 19	≥ 20	Cytoreductive surgery and hyperthermic intraperitoneal chemotherapy	NR	NR	1 mg/kg over 20 min, followed by 0.5 mg/kg/h	Saline	KDIGO
Sun et al. (33)	Dex: 28 Control: 28	18–65	Laparoscopic colorectal cancer surgery	midazolam 2.0 mg, propofol 1.5 mg/kg, cis-atracurium 0.2 mg/kg, fentanyl 3.0 μg/kg	Sevoflurane 1.5–2%, cis-atracurium 5–10 mg, remifentanil 0.2–0.3 μg/kg/min	1 μg/kg for 10 min, followed by of 0.5 μg/kg/h	Saline	KDIGO
Tang et al. (11)	Dex: 38 Control: 37	20–70	Cardiac surgery	Etomidate 0.3 mg/kg, sufentanil 0.6 μg/kg, rocuronium 0.9 mg/kg	Sufentanil, propofol, sevoflurane 1–2%	1 μg/kg for loading does, followed by 0.3 μg/kg/h	Saline	KDIGO
Wang et al. (36)	Dex: 326 Control: 326	≥ 18	Cardiac surgery	Midazolam, sufentanil, etomidate	Propofol, sufentanil, sevoflurane	0.6 μg/kg for 10 min, followed by 0.4 μg/kg/h	Saline	KDIGO
Wang et al. (16)	Dex: 22 Control: 22	adult	Hepatectomy	Propofol, fentanyl 3 μg/kg, cis-atracurium 0.2 mg/kg	Propofol, fentanyl 1–2 μg/kg, cis-atracurium 5–10 mg boluses	1 μg/kg over 10 min, followed by 0.3 μg/kg/h	Saline	NR
Wu et al. (29)	Dex: 44 Control: 45	60–79	Laparoscopic radical prostatectomy	midazolam 0.05 mg/kg, sufentanil 0.3–0.4 μg/kg, propofol 1.5–2.0 mg/kg, cis-atracurium 0.2 mg/kg	Remifentanil, sevoflurane, cis-atracurium	1 μg/kg for 10 min, followed by 0.5 μg/ kg/h	Saline	KDIGO
Xing et al. (35)	Dex: 309 Control: 310	≥ 60	Noncardiac surgery	Propofol and sufentanil, with or without inhalation of a 1:1 nitrous oxide-oxygen mixture		0.6 μg/kg for 10 min, followed by 0.5 μg/kg/h	Saline	KDIGO
Zhai et al. (26)	Dex: 36 Control: 36	18–75	Cardiac surgery	Midazolam 0.02 mg/kg, sufentanil 0.6 μg/kg, etomidate 0.2 mg/kg	NR	0.6 μg/kg for 15 min, followed by 0.2 μg/kg/h	Saline	RIFLE

Dex, Dexmedetomidine; AKI, Acute kidney injury; NR, not report; RIFLE, Risk, Injury, Failure, Loss, End-stage renal disease classification; AKIN, Acute Kidney Injury Network; KDIGO, Kidney Disease Improving Global Outcomes, ICU, Intensive Care Unit.

### Risk of bias

**Figure 2:**
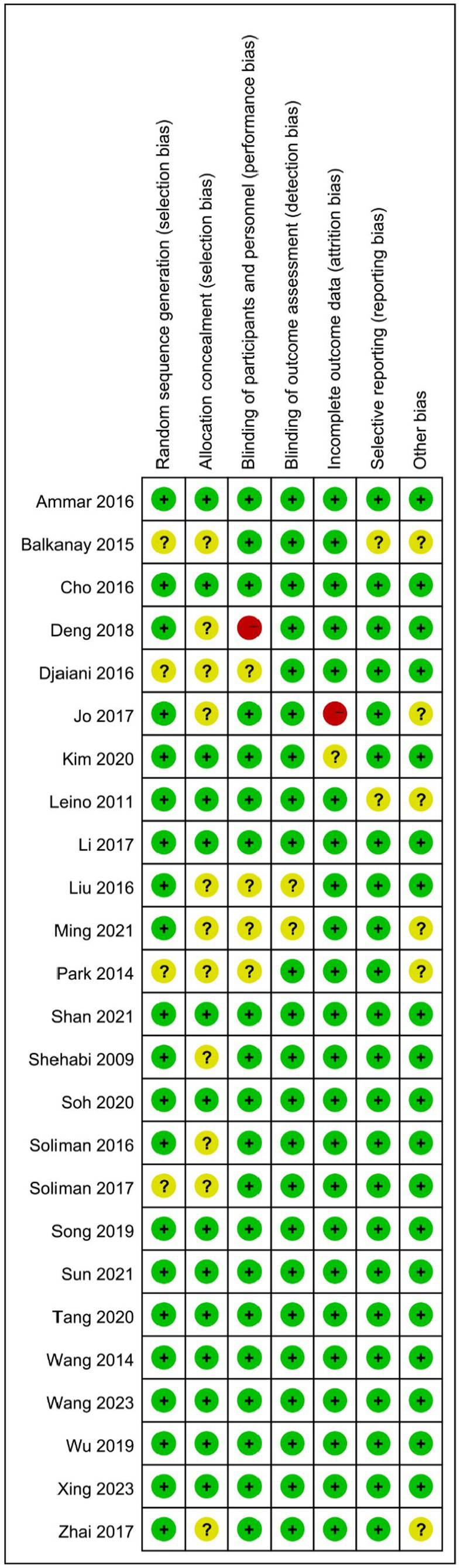
A summary of risk bias for eligible trials.

Figure 2 provides an overview of bias risk across the comprised investigations. Four investigations lacked clear reporting of the randomization procedure (15, 17, 20, 25). Eleven trials did not specify whether allocation methods were blinded (13, 15, 17, 20–23, 25–27, 32). Five trials did not adopt double blinding (15, 20, 21, 27, 32). Two studies indicated that the individuals responsible for evaluating the outcomes were aware of the treatment assignments (21, 32). Furthermore, two studies were identified as having a potential risk of missing outcome data (23, 30), and two trials had a risk of selective reporting (14, 17).

## Meta-analysis

### Primary outcomes

**Figure 3:**
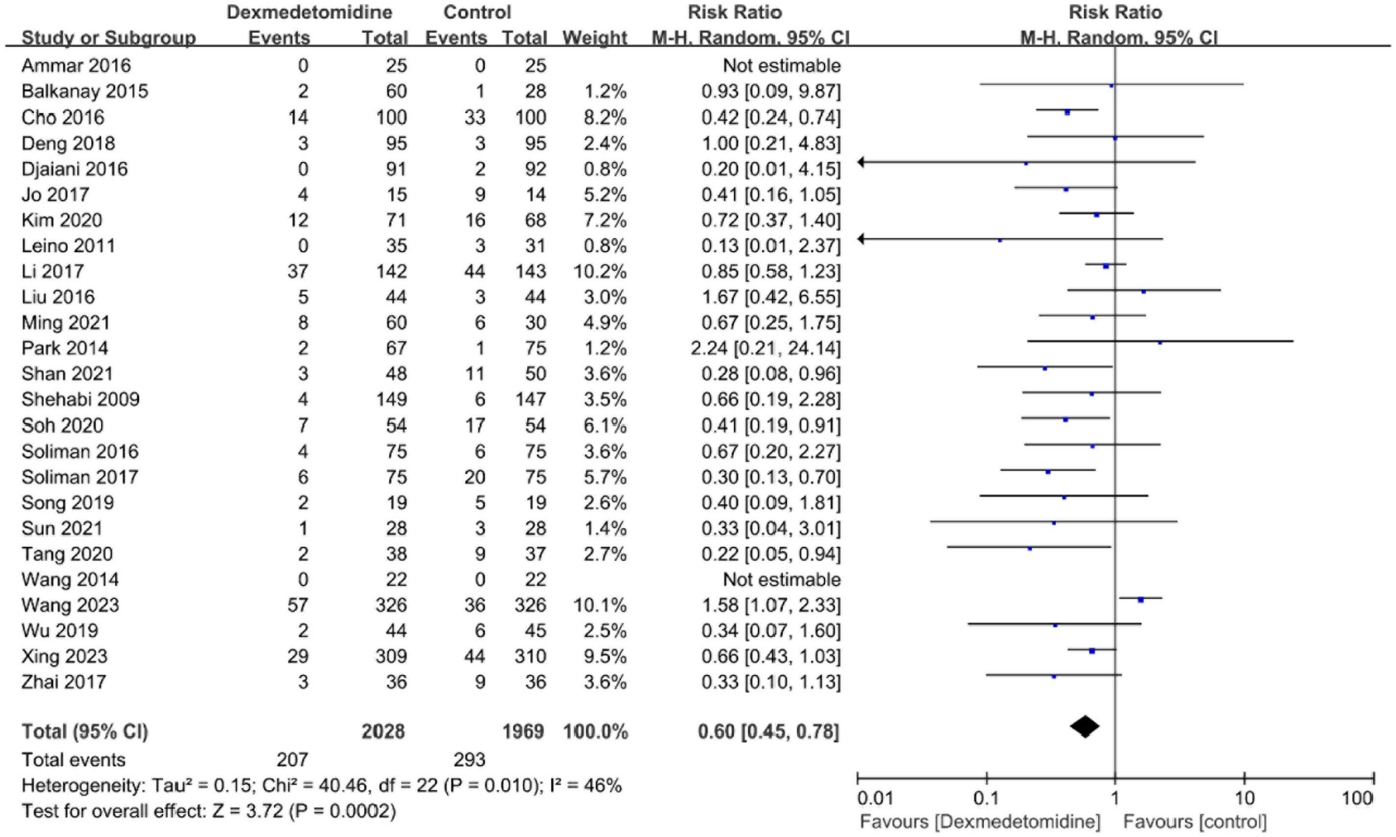
A forest plot showing the occurrence of AKI between two groups. AKI, acute kidney injury.

The POD incidence was evaluated in all trials. The forest plot analysis revealed a significant reduction in AKI occurrence among individuals who were administered dexmedetomidine, in contrast to the control group (RR, 0.60; 95% CI, 0.45–0.78; *p* < 0.05; *I*^2^ = 46%, Figure 3). Furthermore, we performed subgroup analyses depending on various kinds of surgeries (cardiac vs. non-cardiac surgery, Supplementary Figure 1) and different age groups (children vs. adults, Supplementary Figure 2). The findings demonstrated that dexmedetomidine did not have a statistically significant influence on the occurrence of AKI in subjects having non-cardiac operations.

### Secondary outcomes

**Figure 4:**
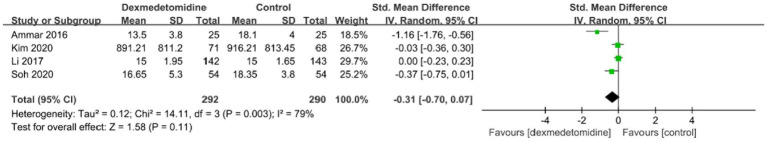
A forest plot showing the mechanical ventilation time between two groups.

**Figure 5:**
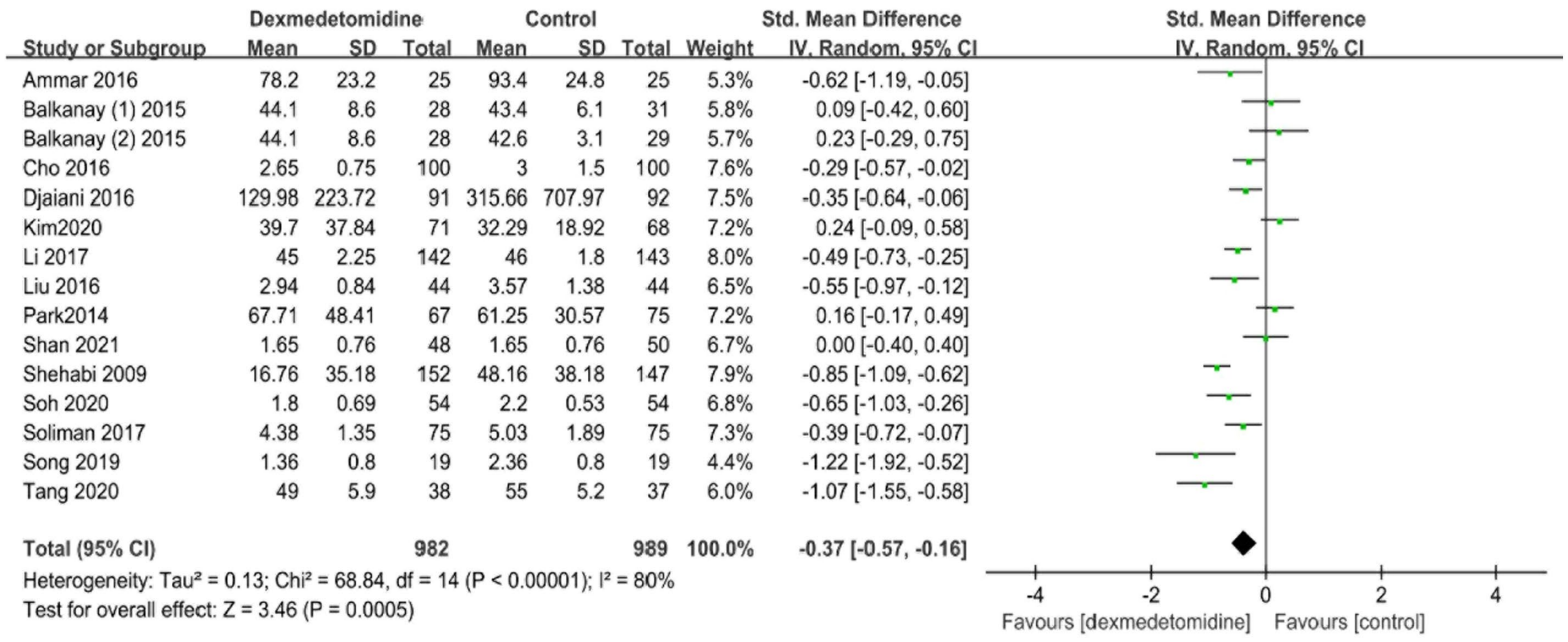
A forest plot showing the duration of stay in the ICU between two groups. ICU, intensive care unit.

**Figure 6:**
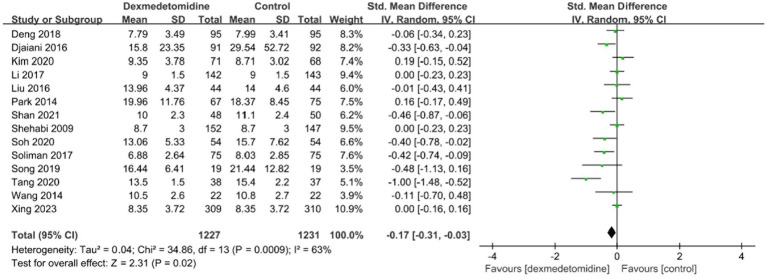
A forest plot showing the duration of stay in the hospital between two groups.

Within the trials that were included, four investigations evaluated the time length of being connected to a mechanical ventilator. The meta-analysis found no statistically significant variation in the duration of mechanical ventilation between the dexmedetomidine and control groups (standardized mean difference [SMD], −0.31; 95% CI, −0.70–0.07; *p* = 0.11; *I*^2^ = 79%; Figure 4). Fourteen trials provided data on the period of hospitalization in the ICU and hospital. The forest plot analysis exhibited that dexmedetomidine significantly diminished the duration of presence in the ICU (SMD, −0.37; 95% CI, −0.57–-0.16; *p* < 0.01; *I*^2^ = 80%; Figure 5) and hospital (SMD, −0.17; 95% CI, −0.31–-0.03; *p* < 0.05; *I*^2^ = 63%; Figure 6).

**Figure 7:**
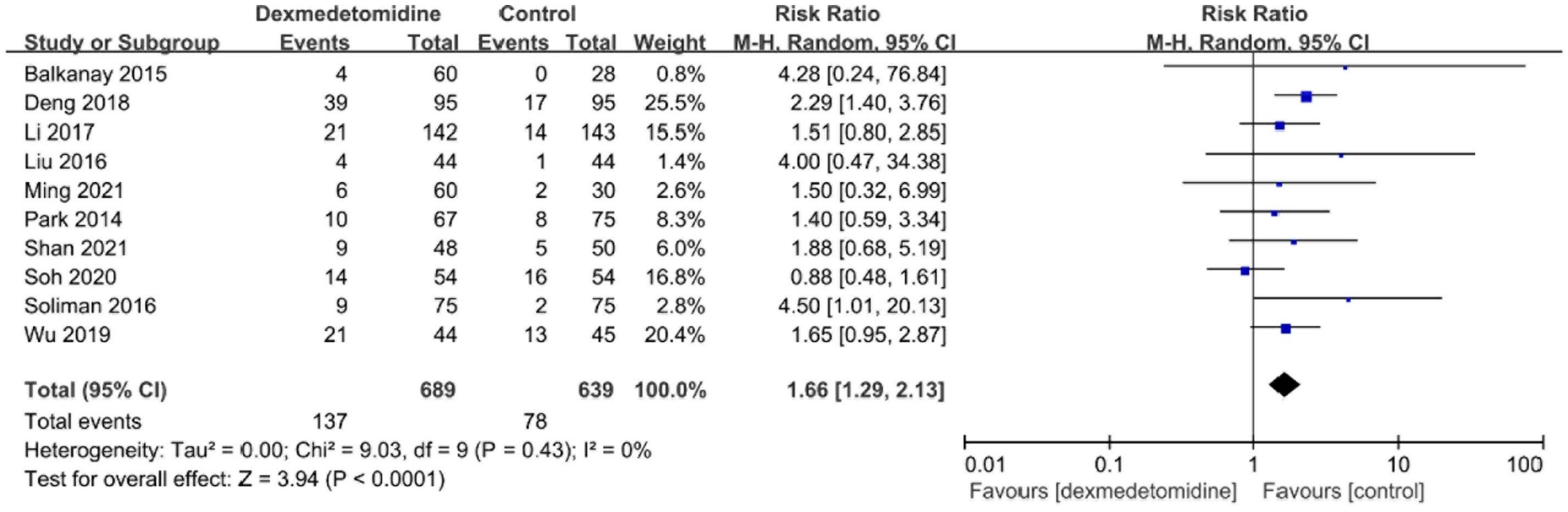
A forest plot showing the occurrence of bradycardia between two groups.

**Figure 8:**
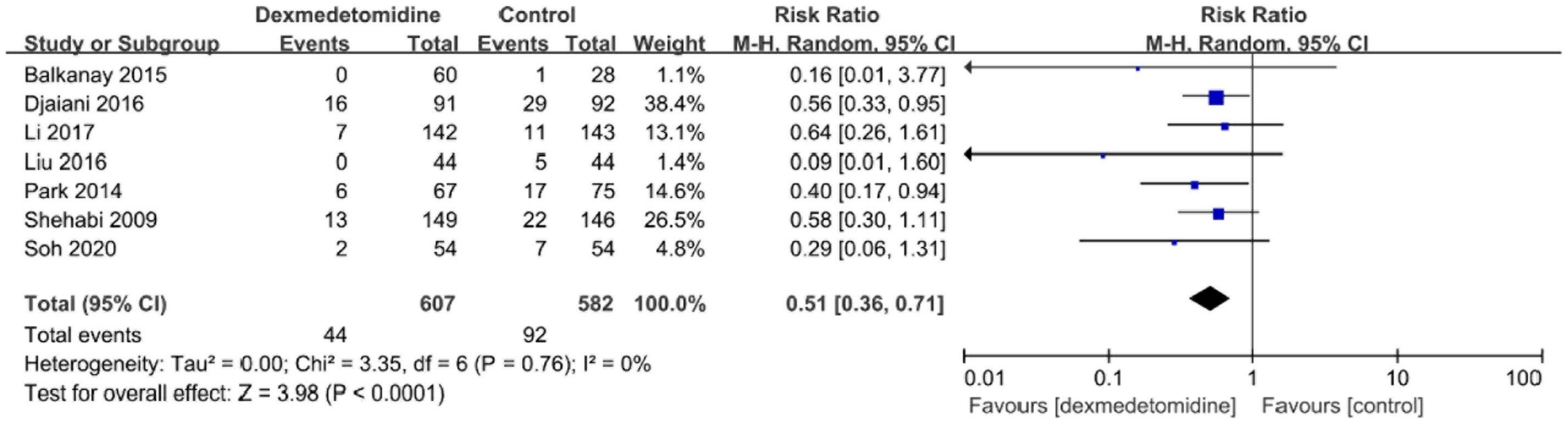
A forest plot showing the duration of POD between two groups. POD, postoperative delirium.

The occurrence of bradycardia was examined in ten investigations, and the forest plot revealed that individuals who received dexmedetomidine had a much higher probability of experiencing bradycardia in contrast to the control group (RR, 1.66; 95% CI, 1.29–2.13; *p* < 0.05; *I*^2^ = 0%; Figure 7). POD occurrence was observed in seven investigations, and the forest plot demonstrated a significant decline in the frequency of POD with the treatment of dexmedetomidine (RR, 0.51; 95% CI, 0.36–0.71; *p* < 0.05; *I*^2^ = 0%; Figure 8). Furthermore, the meta-analysis findings indicated that there was no statistically significant variation between both groups in relation to the occurrence of death, stroke, or low blood pressure (Supplementary Figures 3–5).

### Publication bias

The funnel plot for the primary outcome showed a symmetrical distribution on both sides (Supplementary Figure 6), indicating no apparent publication bias. Additionally, Begg’s test yielded a value of 0.874, further supporting the absence of significant publication bias.

### Trial sequential analysis

The trial sequential analysis (TSA) conducted to evaluate the prevention of AKI with dexmedetomidine exhibited that the cumulative z-curve reached both the standard and TSA borders. This suggests that sufficient evidence has been accumulated and that no further investigations are necessary to verify the effectiveness of dexmedetomidine in preventing AKI (Supplementary Figure 7).

### GRADE result

The primary outcome was evaluated as having a high level of evidence quality, whereas the evidence quality for other outcomes varied from low to high. Table 2 shows detailed data on the GRADE assessment.

**Table 2:** The summary of GRADE.

Outcome	Included studies (*n*)	Patients (*n*)	Quality of evidence	Reasons
Incidence of AKI	25	3,997	⨁⨁⨁⨁ HIGH	None.
Duration of mechanical ventilation	4	582	⨁⨁⨁◯ MODERATE	“Inconsistency” was downgraded to “serious.”
Duration of ICU stay	14	1943	⨁⨁⨁◯ MODERATE	“Inconsistency” was downgraded to “serious.”
Duration of hospital stay	14	2,458	⨁⨁⨁◯ MODERATE	“Inconsistency” was downgraded to “serious.”
Incidence of bradycardia	10	1,328	⨁⨁⨁⨁ HIGH	None.
Incidence of POD	7	1,189	⨁⨁⨁⨁ HIGH	None.
Incidence of mortality	9	1,499	⨁⨁⨁⨁ HIGH	None.
Incidence of stroke	6	924	⨁⨁⨁⨁ HIGH	None.
Incidence of hypotension	8	1,131	⨁⨁⨁◯ MODERATE	“Inconsistency” was downgraded to “serious.”

GRADE, Grading of Recommendations Assessment, Development, and Evaluation; AKI, acute kidney injury; ICU, intensive care unit; POD, postoperative delirium.

## Discussion

Our meta-analysis, which included 25 RCTs, provides strong evidence that perioperative administration of dexmedetomidine has potential renal protective effects and significantly decreases the incidence of AKI following the operation. The quality of the evidence supporting these results is good. Furthermore, dexmedetomidine treatment was connected to shorter lengths of hospitalization in the ICU and hospital, in addition to a lower incidence of POD. However, it is vital to note that the incidence of bradycardia is elevated with dexmedetomidine use.

AKI is a common and significant complication that happens following surgery. In our study, the overall incidence of AKI was 12.5%, which was basically consistent with the previous research results. Kheterpal et al. conducted an investigation of 152,244 individuals who had general surgical procedures and found various risk factors leading to AKI, such as older age, being male, undergoing emergency or intraperitoneal surgery, having diabetes mellitus that requires oral or insulin treatment, having active congestive heart failure, having ascites, having hypertension, and having varied degrees of preoperative renal insufficiency (37). The formation mechanism of AKI is complex and involves multiple factors during the perioperative period, such as reduced renal blood flow, contrast-induced nephropathy (38, 39), and inflammation and stress response connected to surgery (40).

Dexmedetomidine, widely utilized in medical anesthesia for sedation, analgesia, and general anesthesia, has shown renal protective effects in animal experiments by reducing the inflammatory response and ischemia–reperfusion renal injury (41). Yang et al. found that dexmedetomidine could inhibit oxidative stress and prevent AKI by affecting the NOX4/Nrf2/HO-1/NQO1 signaling pathway (42). Zheng et al. believe that dexmedetomidine may reduce the occurrence of AKI through NLRP-3/caspase-1 mediated pyroptosis (43). Some clinical trials have also suggested a reduced incidence of postoperative AKI with perioperative administration of dexmedetomidine (36, 44). However, there is ongoing debate related to the efficiency of dexmedetomidine in AKI prevention. A new meta-analysis and two high-quality RCTs reported no significant influence of dexmedetomidine on the AKI incidence (45). Therefore, after conducting a systematic search, we performed this meta-analysis that included 25 RCTs. The results of our meta-analysis reveal that the usage of dexmedetomidine during the perioperative period significantly decreased the AKI incidence. Furthermore, the evidence supporting this conclusion was of high quality. TSA also indicated that the number of trials was sufficient to confirm this evidence. Notably, subgroup analysis demonstrated that dexmedetomidine did not decrease the occurrence of AKI among individuals who were having non-cardiac operations. This could be linked to the relatively lower incidence of AKI in non-cardiac surgery patients compared to cardiac surgery. Our investigation revealed a variation in AKI incidence among several surgery categories within the control group (17.1% in cardiac surgery vs. 11.8% in non-cardiac operation). It is vital to know that the sample size of the meta-analysis for non-cardiac surgery was small, indicating the need for further studies to confirm this result.

Our meta-analysis conclusively exhibited that usage of dexmedetomidine led to a significant decrease in the duration of ICU and hospital stays, along with a decrease in POD incidence. These results align with an additional investigation that has advocated for the usage of dexmedetomidine to avoid POD, especially in older patients (46–49). The decrease in the duration of hospitalization in the ICU and hospital can be related to the reduced occurrence of complications following surgery, such as AKI and POD. By mitigating these complications, dexmedetomidine contributes to faster recovery and a shorter duration of stay in hospitals.

Regarding the increased incidence of bradycardia associated with administration of dexmedetomidine, it is a well-known side effect of this drug. Dexmedetomidine acts as an α2-adrenoceptor agonist, which can lead to bradycardia. Therefore, the increased bradycardia incidence observed in our meta-analysis was expected and is a known effect of dexmedetomidine use. Further, our analysis found that dexmedetomidine did not raise the incidence of hypotension. Notably, dexmedetomidine did not raise the incidence of hypotension. This indicates that the use of the recommended dose of dexmedetomidine will not cause significant hypotension, which in turn prevents a reduction in renal blood flow and impairs renal function.

It is worth noting that this meta-analysis includes several kinds of surgeries comprising cardiac and non-cardiac surgeries. Although we conducted a subgroup analysis, heterogeneity did not significantly decrease. Anesthesia induction and maintenance are not standardized, and there are also differences in the timing and dosage of dexmedetomidine use. In addition, there is a lack of unified standards for the definition of AKI. Considering the various factors mentioned above, which led to high clinical heterogeneity, we adopted a random-effects model.

Some limitations of this study should be pointed out. First, due to a lack of data, we did not conduct a subgroup analysis depending on different administration times and doses of dexmedetomidine. Second, multiple factors lead to a certain degree of clinical heterogeneity in this study, which requires further confirmation from higher quality and homogeneous clinical studies. Third, the sample size for non-cardiac surgery was small, requiring further confirmation. Fourth, we did not analyze the matching risk factors of age, diabetes, hypertension, etc. between dexmedetomidine and the control group. Furthermore, no further subgroup analysis was conducted on specific surgical types.

## Conclusion

In conclusion, our meta-analysis demonstrated that dexmedetomidine can effectively decrease the risk of AKI and POD following cardiac operation, in addition to shortening the period of ICU and hospital staying. Dexmedetomidine should be promoted but further investigation into the types of surgeries where it benefits need to be investigated.

## Author contributions

JZ: Writing – original draft, Software, Methodology. M-hT: Writing – review & editing, Supervision. Q-hS: Writing – review & editing. D-cX: Data curation, Writing – review & editing.
